# Experiences from cross-cultural collaboration in health campaigns in Tanzania: a qualitative study

**DOI:** 10.1186/s13690-021-00730-0

**Published:** 2021-11-17

**Authors:** Olav Johannes Hovland, Ane Falnes Hole, Mercy Grace Chiduo, Berit Johannessen

**Affiliations:** 1 Department of Health and Nursing Sciences, Faculty of Health and Sports Sciences, University of Agder, Building I, Postbox 422, 4604 Kristiansand, Norway; 2 National Institute for Medical Research, Tanga Medical Research Centre, P. O Box 5004, Tanga, United Republic of Tanzania

**Keywords:** Public health, Health promotion, Health information, Health literacy, Health campaigns, Cross-cultural collaboration, Tanga international competence Centre (TICC), Qualitative study

## Abstract

**Background:**

Health campaigns are an important aspect of preventive health work. They can aim to improve health literacy in rural areas where residents lack access to health information and knowledge, and to improve both local and global health through cross-cultural collaboration. In Tanga District, Tanzania, exchange students and local youths participate together with Tanga International Competence Centre (TICC) to plan and accomplish health campaigns in local communities. The aim of this study was to explore the participants’ experiences with the cross-cultural collaboration in the planning and delivery of TICC’s health campaigns.

**Methods:**

This study used a focused ethnographic approach. Five weeks of fieldwork included four observations of health campaigns and nine interviews: three individual interviews with employees at TICC (all Tanzanians), two group interviews with nine Norwegian nursing students, two group interviews with five local youths enrolled in TICC’s Youth Program, one interview with a local village leader, and one interview with a local primary school teacher. The interview material was analyzed using systematic text condensation.

**Results:**

All participants experienced the cross-cultural collaboration as successful. Having enough time, adapting to local conditions, and understanding the needs of the target groups were perceived as essential to the campaigns’ success. Music and role-play, which are dominant within Tanzanian culture but not common among the Norwegian students, created excitement and motivation among the audiences. The interviewees identified changes in people’s health behavior in the aftermath of the campaigns.

**Conclusion:**

All participants in this study identified positive outcomes from the cross-cultural collaboration within TICC’s health campaigns. The health campaigns were considered beneficial because of the poor access to health information among residents in the local communities.

## Background

Health information is an important tool in preventive health work, whose aim is to influence a population’s behavior as well as to strengthen the individual’s motivation and to provide opportunities to make health-promoting choices [1]. According to Nutbeam [2] and Nutbeam, McGill and Prekumar [3], a person’s health competence includes a focus on autonomy and empowerment, and this definition places health information within a health-promoting approach that emphasizes individual opportunities and personal resources.

In an increasingly globalized world, cross-cultural communication and collaboration are often used in preventive health work. Cross-cultural refers to the comparison of phenomena across cultures [4]. An integral part of a cross-cultural relationship is understanding how the interactions with people from different cultures may be unlike the interactions accustomed to one’s own culture [5]. Cultural differences are fundamental, and collaborative and synergistic relationships require cooperation and an appreciation of cultural differences [5].

Cross-cultural collaboration between individuals and communities is crucial for achieving common goals and creating varied and multiple solutions to health problems. The local community is a key factor that influences the development of a person’s health and health behavior [6]. The local community can be a strategic arena for promoting health in which efforts are directed towards increasing people’s influence and control over conditions that affect their health [1, 7]. The interplay between the social network, life events, and external conditions can affect health [8], and the local population can therefore be considered an active partner in local health work [9]. The main goals in health promotion are to facilitate collaborative decision-making to solve common problems and to stimulate the local community to action [7].

The use of communication strategies that reflect the target group’s social and cultural reality, as well as using tools or devices that appear to be relevant and appealing to the target group, can promote the target group’s receptivity to the message [10–12]. This may help contribute to the target group’s recognition of the topics presented in the campaign and provide the context for ensuring that health information is perceived as comfortable and safe [11].

Conveying information with a focus on understanding the target group’s cultural framework and socioeconomic status can be crucial to the success of programs in disseminating health information [9]. Studies have shown that health campaigns are effective in improving rural people’s knowledge and attitudes about different health issues [13, 14]. Statistics from 2019 (Table 1) show that Tanzania has a high student dropout rate and that many students do not continue their education after primary school [15]. This low educational level can influence the level of health literacy.

**Table 1:** Total Enrolment (number) by Level, Tanzania Mainland, 2019

Level	2019 (Total)
Primary	10,605,430
Secondary Forms 1–4	2,185,037
Secondary Forms 5–6	153,420
Secondary Forms 1–6	2,338,457

Source: Ministry of Education, Science, Technology and Vocational Training of Tanzania, http://www.moe.go.tz/en

The current study focuses on cross-cultural collaboration between local participants and staff in a health campaign program (HCP) and Norwegian nursing students in Tanzania. One of the aims of the HCP is to improve the knowledge about health issues in rural communities.

### Context

The study was performed at Tanga International Competence Centre (TICC) [16] in the Tanga region, Tanzania. TICC is a nongovernmental and nonprofit organization founded by Norwegians in 2008. The center’s vision is to connect (people), combine (knowledge), and create (new opportunities), with the aim of improving health and education for the residents of the Tanga region through cross-cultural collaboration with local and regional authorities and partners. TICC operates seven social programs in neighboring communities and invites the Norwegian exchange students to complete parts of their studies in one or more programs. At the start of the exchange, the students undertake 1 week of Kiswahili language instruction and introduction to the Tanzanian health system and culture, after which they participate in programs focusing on elderly people, children, mental health, school health, and community awareness and campaigns, each lasting 2 weeks. Employees at TICC supervise the students in their clinical placement and act as interpreters between the patients and students.

Since 2008, Norwegian nursing exchange students have visited TICC for 3-month clinical placements. From 2008-2011, many of these students spent 6 weeks or more living with a local family in one of the nearby villages. During their stay, the students were given an assignment to map the health challenges of the village residents. These mapping projects in the villages around Tanga city revealed a low level of health literacy among the residents, especially regarding basic knowledge of nutrition, the need for daily intake of water, personal hygiene, contamination control, mental health, malaria prevention, sex education, and the importance of keeping the surrounding areas clean. In 2011, TICC discussed the mapping results with the village chiefs and residents. The main questions discussed were as follows: (i) What is your main health challenge or issue? (ii) What can you do yourself? (iii) What can we do? (iv) Who can do something? and (v) How should the plan be implemented? One of the results of this cross-cultural communication process between TICC and the local communities and regional authorities in Tanga was the decision to initiate health campaigns in the villages. After interactions and more discussions with the local communities, the HCP also included visits to the local primary and secondary schools.

The HCP aims to convey information and teach local communities various topics that are relevant to people’s health and livelihoods. Some topics that have been central to the HCP are the importance of basic hygiene, dental health, access to and the daily need to drink enough clean water, mental health and stigma, issues related to the human immunodeficiency virus and acquired immunodeficiency syndrome, malaria, bullying, and drug addiction. The topics of the campaigns are identified through cross-cultural collaboration between the Norwegian nursing students and the local employees at TICC and are usually structured as follows. First, a short oral introduction is given by one of the staff from TICC, followed by a lecture given by Norwegian nursing students in English and translated into Kiswahili, Tanzanian’s national language spoken and understood by most people. The lecture is followed by music, dance, and song containing the health message, after which local youth enrolled in TICC’s Youth Program perform a play in Kiswahili that is related to the health message. At the end, the audience is asked questions, and gifts are given to those who can provide the right answer. In turn, the audience can ask questions of the students, youth, or TICC staff. The whole performance lasts about 90 min.

The aim of this study was to explore the participants’ experiences with the cross-cultural collaboration in the planning and delivery of TICC’s health campaigns.

## Methods

### Study approach

This study used a focused ethnographic approach. According to Cruz and Higginbottom [17], focused ethnography emphasizes the description of cultural behavior, through which one learns about people by learning from them. This study was time limited and addressed a predefined research question, and a strategic sample design was used. The participants were assumed to have specific knowledge or experiences of interest to the project. The data material was obtained during 5 weeks of fieldwork.

### Sample and setting

Four groups were identified as the desired sample: Tanzanian employees of TICC who were affiliated with the HCP, Norwegian nursing exchange students who had participated in the HCP as part of their 12-week internship at TICC, participants in TICC’s Youth Program who had taken part in the HCP, and TICC’s contacts in the local community who held key positions at relevant schools (schoolteacher) and villages (village leader) where the campaigns were held.

All invitations to participate in the interviews were provided by staff at the Study Section at TICC. Written information and consent forms were prepared in Norwegian, English, and Kiswahili, and these were given to a research assistant from TICC who distributed the forms to the participants. Oral information was given in advance of the interviews. The information noted that all data were depersonalized and that participants had the right to withdraw from the study at any time. The data material contained no codes or other personal identification. After the transcriptions were completed, all audio records were deleted. All files were saved on the university’s server, which required a two-step identification method to obtain access.

Semistructured interview guides were designed for the different participant groups. The interview guides focused on the experiences of the cross-cultural collaboration in the planning and delivery of the campaigns.

### Interviews and observations

Nine interviews and four observations of health campaigns were conducted: three individual interviews with the employees responsible for the HCP at TICC (all Tanzanians), two group interviews with nine Norwegian nursing students participating in the HCP at the time the study was performed, and two group interviews with five local youths enrolled in the TICC’s Youth Program. We also performed individual interviews with a local village leader and a local primary schoolteacher at the school where one of the campaigns was conducted during the study. Because some of the Tanzanian informants did not speak English and the researchers did not speak Kiswahili, five of the interviews were interpreted by a Kiswahili-speaking research assistant. All interviews were performed by author AFH.

Observations can strengthen the understanding of the health campaigns and the cultural context and can provide opportunities for the researcher to become immersed in the culture studied. Three of the campaigns observed in this study dealt with substance abuse; two took place in secondary schools and one in a village. The fourth observation was of a campaign focused on malaria that was held in a primary school. The observations aimed to collect background information and to provide understanding of the organization and implementation of the campaigns. The background information obtained from observations was not further analyzed.

### Analysis

The interview material was audio recorded and transferred to written form through a transcription process [18]. The transcribed text material was entered into NVivo12® (QSR International, Melbourne, Australia) for further organization and analysis. The text transcribed from the interviews was analyzed using systematic text condensation, an inductive approach, in four steps [18].

1. Reading all the material to obtain an overall impression and noting tentative themes;
2. Identifying units of meaning and coding different aspects of the participants’ experiences;
3. Condensing and abstracting the meaning within each of the coded groups;
4. Summarizing the contents of each coded group to generalize descriptions and concepts to main themes reflecting the participants’ most important experiences.

Translation of interviews and quotes can be challenging because of the difficulty in translating culture-specific words. We performed the interviews in both Kiswahili translated to English and in English. During the interview preparations, author AFH presented the themes in the interview guide and the aim of the study to the interpreter and discussed the importance of consistency in the translation and how to perform the interviews. To ensure correct interpretation and proximity to the material, it is important to stay with the original language for as long and as much as possible [19]. Therefore, the interviewer asked follow-up questions to confirm information or to obtain further descriptions of the participants’ experiences. Because transcriptions filter the material, AFH transcribed all interviews to retain the same style and structure of the transcription process [20]. She listened to the records, transcribed them, and returned to the records to confirm the content and meaning in the interviews. Notes were taken during the interviews and were used to support the transcription process. Using more words than stated in the original quotes or interpretations can also change the informant’s voice [19]. Despite these challenges of translation and interpretation, the translated quotes are presented in the Results section.

To enhance and strengthen the trustworthiness of the material, author AFH performed the analysis following steps (1)–(3) above and discussed the findings with authors OJH and BJ; all three authors worked together to summarize the content during step (4). Table 2 displays an overview of the main themes.

**Table 2:** An overview of the main themes

Norwegian nursing students	Participants in the TICC Youth Program	Employees of TICC	Local schoolteacher and village leader
1. There is a need for cultural guidance and collaboration.	1. The collaboration with Norwegian nursing students is valued.	1. Cross-cultural collaboration ensures the quality of the campaigns.	1. The cross-cultural campaigns are valued.
2. Thinking alternatively and creatively helps to reach the audience.	2. Local adaptation is crucial to reaching a selected target group.	2. Adaptation to local conditions is crucial to reaching and benefiting the audience.	2. The campaign presentations are engaging.
3. Participating in campaigns promotes achieving the learning outcomes.	3. Joining cross-cultural campaigns is empowering.	3. Cross-cultural campaigns motivate people’s interest in health information.	3. Acquiring health information changes people’s everyday lives.

## Results

The results showed that cross-cultural collaboration was perceived as beneficial by all participants and that the use of an accommodated communication strategy was essential to fulfilling the purpose of the health campaigns. The participants highlighted the campaigns’ relevance for increasing the level of health literacy among the residents in the local communities.

### Experiences of the Norwegian nursing students

The nursing students said that working in a Tanzanian context is very different from the Norwegian one, and they regarded guidance by the responsible staff at TICC as necessary. Supervision by and cooperation with employees at TICC was considered crucial for deciding which topics were relevant to present. Collaboration with an interpreter was necessary. However, the use of interpreters was also linked to uncertainty and lack of control over what and how the information was communicated to the public. Several students expressed concerns about misinformation in relation to the interpreter’s prior knowledge of the topic, which was perceived as crucial in determining how the information was translated.

It was considered important to adjust the campaigns’ themes and information to gain acceptance within the local culture. The students said that they had to think creatively to be able to present controversial topics without offending anyone or conflicting with the cultural norms. Several highlighted how music, song, and dance are a central part of Tanzanian culture and that their incorporation into the campaigns was useful for capturing the audience’s attention. The play was considered important for relating the information to everyday situations. The students also perceived that meeting people where they live their daily lives was necessary for reaching the target group. One of the students said, “*Here you actually have to go out to meet people. You cannot just write it on a blog or an online newspaper*.”

The students discussed their experiences of mastery by successfully implementing the campaigns. Some mentioned their uncertainty about the relevance of the campaigns in relation to their academic benefits. Others said that the campaigns were perceived as an exercise and part of their training to fulfil a pedagogical function. They valued the learning outcomes from attending the health campaigns. Several of the students talked about the responsibility of the health professional when disseminating health information. Fear of giving incorrect information when answering questions from the audience and the pressure to keep to the schedule were expressed as concerns because the topics presented in the health campaigns were decisive to the future health choices of the attendees.

### Experiences of participants from the TICC youth program

The local youths enjoyed collaborating with the Norwegian nursing students and perceived the students as pleasant, cooperative, and welcoming; these feelings were considered to be mutual. The informants felt that their contribution to the health campaigns was valued and respected by the students. Several youths mentioned that they gained knowledge through their collaboration with the students. They considered this important for their life and health situation. “*The students strengthen us. They let us talk to them, and they let us learn through the campaigns we perform together*.”

The youths specifically mentioned that campaigns are an appropriate method to reach out to people in the villages that lacked access to electricity.

*If you travel to villages without electricity, they [the inhabitants] can also, through the use of campaigns, get information. They would not have received this information if it was communicated via radio or television, since they do not have electricity. But with campaigns, because they see you physically, they can learn in a reasonable way.*

Using different methods to disseminate the health information was perceived as essential. One commented, “*I like the way we present the information. Some can understand through words, while for others the information becomes clearer through acting, singing, and dancing*.”

The campaigns gave the youths opportunities to share their experiences with others who sought their expertise, and they talked about their increased self-confidence and self-esteem as a result of their involvement in the HCP. One noted, “*The campaigns give us strength, self-confidence, and help us trust ourselves so that we can stand in front of many people and teach*.”

The local youths described the health campaigns as sustainable because each campaign’s audience communicated what they had learned to others who were not present. They perceived that the audience appreciated and enjoyed the campaigns, and that the local community learned much.

### Experiences of employees at TICC

The employees described the Norwegian nursing students as committed and open minded about doing something new. Both the nursing students’ learning goals and the population’s benefits were perceived as important priorities. Given the different cultural and professional orientations, it was sometimes challenging to understand what the students presented in the planning phase of the campaigns. Nevertheless, the students’ competence was considered to be important to the academic quality of the health campaigns. One employee said, “*When we travel and the students are with me, I feel very safe and confident*.”

The collaboration with the youths enrolled in the TICC youth program was perceived as important, and it was considered favourable that the play was presented in Kiswahili. They perceived that the youths were positive and willing to do something new. One employee said about the youths, “*For many, I think it is like stepping out of their own comfort zone. One tries to do something one has never done before. Many have never acted in plays … It is like opening up a new world for their talents, in a way*.”

The employees said that it was important to investigate whether the students’ chosen topics reflected a local challenge. Their experiences as Tanzanians were presented as an advantage in such assessments. One commented, “*I’m from Tanzania. I live here, I can see the challenges in my community.*”

They said that the final choice of themes for the health campaigns were based on both the students’ interest and the employees’ local insight and competence. This collaboration was important for adapting the content to the area and target group. An employee commented, “*For example, now students have chosen the topic of malaria. It was the right time for this topic, because it rains a lot and then there are a lot of malaria mosquitoes.*”

The employees perceived the campaigns as important for people’s understanding of health-related topics. Schizophrenia was exemplified as a phenomenon that residents believed was caused by witchcraft. After a campaign about schizophrenia, the audience talked about schizophrenia in a different way, and the employees perceived that people understood that it was not connected to witchcraft.

The employees reported uncertainty associated with the long-term effect of the health campaigns. After each campaign, evaluation and reflection were conducted among those involved in the TICC. Conducting the formal evaluations of the campaigns among the audience was challenging, and the employees were uncertain about the best method for this. The employees said that they usually discuss each campaign with the local population to form an impression of the campaign’s usefulness and the audience’s response. Several mentioned that the Norwegian nursing students’ participation in HCP created great audience engagement.

### Experiences of a village leader and a primary school teacher

The village leader said that the actors from the TICC were cooperative and believed it desirable to maintain this relationship. He perceived the Norwegian nursing students’ involvement in the health campaigns as valuable. He commented, “*The kindness that the students bring; they show us great care. People feel that they are valued by the students coming here to meet them*.”

The campaigns were considered as useful, and both the leader and the teacher assumed that, after the campaign, the audience discussed what they had learned with others. The teacher mentioned particularly that her pupils needed health information and that they had not learned about the topics before the campaign presentation. The teacher highlighted that to carry on with the campaigns were important in the work with health information. The teacher thought that the different activities in the campaign enhanced the children’s interest in learning. The village leader said that the use of entertainment was important to create engagement and was something the audience appreciated. Both the village leader and teacher highlighted the use of questions in the end of the campaign as important for the audience’s benefit and that gifts were given to those who answered the questions correctly after the performance. The village leader thought that these gifts contributed to greater attendance and commitment among the audience.

The teacher and the village leader had received feedback from the audiences that the information delivered in the health campaigns was important to them. The teacher mentioned that several parents had thanked her for this initiative. The village leader said, “*People in the village come to me directly and ask for more campaigns. This shows that they like them and that they benefit from them*.”

Both the village leader and teacher had noticed changes in people’s behaviour after the campaigns. The teacher mentioned that she had perceived an improvement in the pupils’ cleanliness after a campaign. The village leader had recently seen, after a campaign about epilepsy, that a family with a child with epilepsy brought the child to the hospital for treatment. The village leader had also noticed changes in people’s behaviour regarding dental health. He said, “*Such things [changes] are a sign that they [the audience] like the campaign, and that they benefit from the campaigns*.”

## Discussion

The purpose of this study was to explore the participants’ experiences with the cross-cultural collaboration in the planning and delivery of TICC’s health campaigns. The HCP participants were both Norwegians and Tanzanians, who had different professional knowledge and cultural backgrounds. All participants in this study identified positive outcomes from the HCPs. The outcomes included responses from the village residents and pupils at primary schools. Given the local circumstances, such as poor access to health information, the health campaigns were considered to be beneficial by those attending the program and the performances.

One criticism of such initiatives is whether exchange students who lack knowledge of and a strong connection to the local culture should be included in local health work. Consideration and respect for cultural perceptions and other ways of problem-solving, dialogue, cooperation with local participants, and the ability to see the opportunities in the local community are crucial for maintaining productive cooperation in such situations [21]. In the cross-cultural planning process of the health campaigns, all people involved bring their cultural perceptions, knowledge, and experiences into the group. This can be challenging, and it is important to acknowledge that inherent conflicts and misunderstandings are an inevitable consequence of cross-cultural relationships [5]. Overcoming these obstacles requires open communication and respect from all the participants. In the students’ curriculum, developing cultural competence is a learning outcome. However, such encounters increase the cultural competence for all involved [22, 23].

Kreuter and McClure [11] note that communication strategies that reflect the target group’s social and cultural reality can promote the target group’s receptivity to the message. To increase the learning outcome for the audience and to minimize misinformation, the students in this study suggested a closer collaboration with the interpreter ahead of the campaign to establish a common understanding of the content in the performance and presentations.

The participants in this study may have had conflicting perceptions of health, and cross-cultural collaboration can shed light on health-related assessments from different perspectives. From a public health perspective, health can be assessed as a holistic concept [24]. This perspective considers health as a complex phenomenon, and inclusion of different disciplines can therefore be important for promoting people’s health. In addition, through cross-cultural collaboration, it is conceivable that those involved in the HCP gained a greater understanding of the concept of health as they worked together to establish a common understanding of the health topics presented in the campaigns.

The Norwegian nursing students had short-term experience with the local culture compared with the local actors, and this difference emphasizes the need for cross-cultural collaboration [24]. On the other hand, the nursing students had evidenced-based knowledge about the health-related topics that were conveyed in the campaigns. Guidance from TICC staff helped them to understand the need for specific culturally appropriate adapted health information in the local community. Because health and illness exist within the cultural environment, adaptation within the context of the cultural environment is essential to the success of health promotion programs [2].

Participants in the HCP used several methods to ensure that the audience truly understood and learned from the campaigns. During the interviews, the informants noted the importance of using music, dance, and acting in the delivery of the campaigns. These were not methods known to the Norwegian students, but they participated because they understood these to be an important part of Tanzanian culture and everyday life [10]. Including these methods in the performance may have contributed to the campaigns’ success and strengthened the audience’s desire and motivation to participate in the campaigns. Participating in the HCP gave the Norwegian nursing students a deeper understanding of and respect for cultural differences, and helped to empower them by requiring them to move outside their comfort zone [21].

Both the local primary schoolteacher and the village leader had noticed changes in people’s health behavior after the campaigns. They had also received feedback from the audiences that the information provided in the campaigns was important and that they appreciated the participation of the Norwegian students. This feedback confirms that cross-cultural collaboration in health campaigns can affect the local community’s health competence and empowerment [6] which, according to Sletteland and Donovan [8], refers to people’s perceived ability to influence their own health and quality of life.

### Limitations

This study collected data from representatives of all participants involved. However, one limitation of our study is that only one village leader and one schoolteacher participated. The researchers had limited prior knowledge of the campaigns, which helped them to maintain a distance from the research. On the other hand, less knowledge may increase the risk of misinterpretation of situations.

Several factors may be interpreted differently in cross-cultural interviews, and different interpretations may affect the relationship between the interviewer and informants. A researcher who spoke Kiswahili and who had a stronger connection to the cultural environment may have used a different approach in the cross-cultural interviews. Cultural factors may also affect the relationship between interviewer and informants. It was therefore important to pay special attention to the formulation of questions, as well as to nonverbal communication, gender, cultural norms, choice of interpreter, and translation. Our awareness of and reflection on the potential for cultural challenges strengthened the quality of the research process. Use of an interpreter can influence the interview material, and it may be difficult to provide a comprehensive translation [25]. Nevertheless, interviews that use an interpreter are considered effective when the interviewers and informants speak different languages [25].

## Conclusion

All participants in this study identified learning outcomes from the cross-cultural collaboration in TICC’s HCPs. The health campaigns were considered beneficial because of the poor access to health information among residents in the local community. The cross-cultural collaboration increased the health knowledge of all involved in this study. The collaboration supported the Norwegian nursing students to gain cultural competence, and the local youths who were involved gained knowledge and were empowered by the collaboration. Employees at TICC were vital for ensuring the quality of the campaigns and in adapting the campaigns to local conditions. The representatives from the local community were appreciative and perceived that the campaigns helped to motivate the residents to change their health behavior. Further research on the effects of cross-cultural collaboration in health-promoting campaigns and their long-term effects is needed.

## Data Availability

All data generated or analyzed during this study are included in this published article.

## Abbreviations

HCP: Health campaign program
TICC: Tanga International Competence Centre
